# On the Pathology and Treatment of Gonorrhœa and Spermatorrhœa

**Published:** 1887-10

**Authors:** 


### Book Reviews.

On the Pathology and Treatment of Gonorrhœa and Spermatorrhœa. By J. L. Milton, Senior Surgeon to St. John's Hospital for Diseases of the Skin, London. Octavo, 484 pages. Illustrated. Price, bound in extra muslin, $4.00. New York: William Wood & Company. 1887.

It is very refreshing to read a medical book which is the product of a vigorous and original mind. Medical writers generally are so hampered by time-honored medical traditions that they do not possess the courage to write a book without dragging in hundreds of alleged facts that have been current for at least half a century. Most of these so-called facts are based on totally false physiological and pathological theories. An author wishes to write a text-book on surgery; he gathers together the works of the fathers in surgery, copies their truths and their errors, writes a few chapters on the germ theory and antiseptics, and lo! he has produced a text-book of modern surgery. Scientific methods of investigation must be applied in medicine, as in chemistry or natural philosophy, in order to make any real advance. Observations should be accurately made and truthfully recorded, and the statements of others should not be accepted unless they rest on the solid basis of experience. In reading Mr. Milton's book, there are several things that at once impress us. In the first place, it is an exceedingly readable book; the author's style is clear and concise, and in nearly every sentence we are conscious of the strong impress of his individuality. In the second place, his independence of thought and the boldness with which he demolishes hoary old traditions attract and then hold the reader's attention. In the third place, the scientific method he adopts in treating his subject makes his book a most valuable addition to medical literature. In the fourth place, the author's honesty pervades the book from back to back. It is everywhere apparent that Mr. Milton is an earnest seeker after truth. The book is not written to bolster up any of the author's hobbies, but to give to the world the results of years of pains-taking and scientific research.

In chapter II., while discussing the pathology of gonorrhœa, a few pages are given by the author to a consideration of the micrococcus peculiar to gonorrhœa, discovered by Dr. Albert Neisser. Mr. Milton has lived to see Salisbury's gonorrhœal fungus and Jusseaume's gonorrhœal alga fall into "innocuous desuetude," and he is therefore very careful not to commit himself as to the part which Neisser's *gonococcus* plays in the etiology and pathology of gonorrhœa.

The subject of the treatment of gonorrhœa furnishes full scope for the author's trenchant satire. He cuts right and left among those who "cure the disease in nearly ninety per cent. of the cases at a single sitting," and those who rush into print with a few cases cured by a new and infallible specific. He points out that the only scientific way of getting at the real value of remedies is to try each one in a large number of cases, and record accurately the duration of the disease in each case. He has followed this method and tested many of the leading remedies used in gonorrhœa. The space of a life-time is not sufficient to thoroughly test all the measures recommended by various authors, and he has, therefore, confined his experiments to such as copaiba, cubebs, diuretics, purgatives and injections.

He evidently does not value copaiba as highly as many of our American writers, for he says on page 76: "I am quite ready to admit that it is of service in a great number of cases, though I myself never had such success with it as some writers have recorded."

Of cubebs he says: "It occasionally cures with marvelous rapidity, but these cases occur in those happily-constituted persons who throw off disorders with extreme ease, and who are freed from any severe gonorrhœa by very simple remedies." He has little faith in turpentine, oil of santal wood and antiphlogistics, but commends the use of aperients, diuretics, local applications of very hot water and injections.

Injections are discussed in a masterly manner. He gives a list containing over seventy-five drugs, which have, at one time or another, been recommended for injection. He has reached the conclusion that the nitrate of silver is the best of all for injections, and next to it the salts of zinc (acetate, chloride and sulphate). When stricture or orchitis follows the use of injections, it is because they have not been properly given. After reviewing many different modes of treatment, he gives at length his own method. This must be read to be appreciated. It is eminently rational and practical.

The complications of gonorrhœa are treated of at great length and many established methods of treatment overthrown by the author: for instance, scalding of urine is treated universally by anodynes, alkalies, demulcents and diuretics. Mr. Milton has tested these different methods systematically, and reached the conclusion that they are all without effect, except diuretics, and their effect is only slight. His treatment for this distressing symptom consists in the "free use of the hot bath and hot bathing to the penis and bladder, moderate abstinence, and the use of no drink but water, tea and very mild diuretics."

All the complications are considered in a manner which proves the author's rich experience and thorough familiarity with his subject. Chapter VII. contains a most excellent article on the pathology and treatment of gleet.

The latter part of the book is devoted to the pathology and treatment of spermatorrhœa. Space forbids an extended criticism of this subject, but the reader will find it fully in keeping with the rest of the book.

